# Development and status quo of digestive endoscopy in China: An analysis based on the national census in 2013 and 2020

**DOI:** 10.2478/jtim-2023-0115

**Published:** 2024-05-21

**Authors:** Yunfei Jiao, Zhiyuan Cheng, Ye Gao, Tianjiao Wang, Lei Xin, Han Lin, Mengxi Cai, Xudong Ma, Zhaoshen Li, Luowei Wang

**Affiliations:** Department of Gastroenterology, Changhai Hospital, Naval Medical University, Shanghai 200433, China; National Digestive Endoscopy Improvement System, Shanghai 200433, China; Department of Gastroenterology, Shanghai General Hospital, Shanghai Jiao Tong University School of Medicine, Shanghai 200240, China; Department of Medical Quality, Medical and Health Administration, National Health Commission of China, Beijing 100044, China

**Keywords:** digestive endoscopy, quality improvement, national census, China

## Abstract

**Background and Objectives:**

Technique and practice of digestive endoscopy are undergoing speedy development all over the world. This study aimed to evaluate its status quo and development in China.

**Methods:**

All hospitals performing digestive endoscopy in mainland China participated in the national census in 2013 and 2020. Retrospective data of hospitals, endoscopists, volumes, and qualities were collected via an online structured questionnaire, and its accuracy and rationality were verified by logical tests and manual reviews. Data from other countries were used to compare with that of China.

**Results:**

From 2012 to 2019, the number of hospitals performing digestive endoscopy increased from 6,128 to 7,470 (1.22-fold), in which primary healthcare played a minor role. The median hospitals per 100,000 inhabitants per provincial region increased from 0.49 (IQR, 0.39-0.57) to 0.55 (IQR, 0.49-0.63). The endoscopists increased from 26,203 to 39,638 (1.51-fold), but their average workload even expanded. Overall volume increased from 28.8 million to 44.5 million (1.55-fold), and most types of endoscopic procedures recorded a high growth rate. Contrastingly, the specific utilization rates were low and paled in comparison with some developed countries. Nationwide, regional utilization rates showed a significant correlation with GDP per capita (*P* <0.001). Overall qualities of digestive endoscopy were excellent, but certain results of quality indicators posed a huge challenge, such as the detection rates of adenoma and early cancers.

**Conclusions:**

Impressive progress has been made in digestive endoscopy with rapidly expanding economy in China. However, primary healthcare, utilization rates, and income-related inequality of regional services were needed to be improved to promote public health better.

## Introduction

Digestive endoscopy enables the diagnosis and treatment of precancerous conditions and early cancers with relatively low invasiveness. Its sufficient use helps to improve patient survival, reduce catastrophic health expenditure, and then benefits the entire population. With the increasing burden of digestive diseases, especially malignancies, digestive endoscopy is in an era of rapid development.^[1,2]^ Globally, its technique and practice vary widely, with developed countries experiencing superiority in resources and development that some have even plateaued.^[3,4,5]^ In contrast, in developing countries, such as Tanzania ^[6]^ Indonesia ^[7]^ and China ^[8,9]^ hu e anana, nonesa, anna, uge gaps and potential growth have always been seen.

Capacity of digestive endoscopy should be adjusted to facilitate both effective screening programs and rigorous quality control required.^[10]^ Surveys and evaluations within a country can help supervise quality improvement and better meet medical needs in health policy and clinical practice. Nevertheless, there were few studies revealing comprehensive information based on the national censuses.

In China, remarkable progress in economy has been made over the past decades. Simultaneously, digestive endoscopy has exhibited drastic changes and progressive growth since its beginning in 1950 s and has a strong foundation with an increasing demand. New cases and deaths of digestive system cancers in China exceeded 1.89 million and 1.47 million per year,^[11]^ respectively. In addition, the disability weight of digestive diseases was found to be high in the Chinese people.^[12]^ Comprehensive data were needed to update for further strategy and more effective utilization. In this study, based on the results from the national census in 2013 and 2020, the current status and development of hospitals, endoscopists, volumes, and qualities of digestive endoscopy in mainland China were analyzed in detail. Moreover, its shortcomings and relevant remedies are discussed.

## Methods

### Census organization

**Figure 1:**
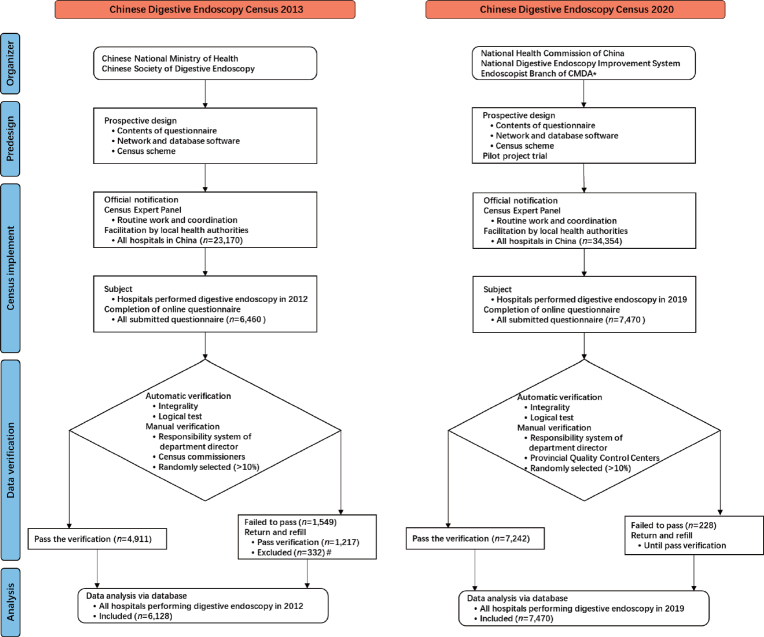
Flow chart of organization of Chinese Digestive Endoscopy Census in 2013 and 2020. *CMDA, Chinese Medical Doctor Association. ^#^The repetitions and the hospitals that had the ability to perform digestive endoscopy but without any volume in 2012 were excluded.

In order to survey and gain a better understanding, the Chinese Digestive Endoscopy Census was designed twice in 2013 and 2020. The overall organization was similar (Figure 1), and the details of the census in 2013 and some of its results have been discussed previously.^[8,13,14]^ In 2020, the census was initiated and undertaken by the National Digestive Endoscopy Improvement System (NDEIS). National Health Commission (NHC) of the People’s Republic of China was responsible for the supervision. An expert panel, comprising authoritative figures from the NDEIS, the NHC, and the Endoscopist Branch of the Chinese Medical Doctor Association (CMDA), was assembled to oversee the routine operations and systematic coordination of this project. All hospitals that performed digestive endoscopy were considered subjects and were invited to participate, which was supported and coordinated by local health authorities.

In the first phase, from September to October 2020, Shanghai was selected as the provincial pilot project to verify the feasibility of the scheme. After the pilot survey, all emerging problems were addressed. During the second phase, from November 2020 to November 2021, the census was rolled out across the whole country, and relevant data were collected.

### Data collection and verification

The census in 2013 and 2020 were completed according to the data from 2012 and 2019, respectively. To submit the information online, exclusive database software was developed. (https://www.ndeis.cn/) To complete a predesigned structured questionnaire, every hospital designated personnel to log into a unique account. In 2020, the census contained more information and three aspects were emphasized in the questionnaire: (1) Information on resources, such as basic information of the hospitals and endoscopists. (2) Utilization of digestive endoscopy, including annual total volume and volumes of different endoscopic procedures. (3) Data of the factors involved in endoscopy qualities.

The accuracy and authenticity of the submitted data were automatically assessed by a logical test using a computer program. Simultaneously, manual reviews were conducted to assess the quality of the original data. A minimum of ten percent of hospitals within each province were subject to random selection by their Digestive Endoscopy Quality Control Centers. Then, corresponding reports were extracted for further check. The rationality of the wholly data in regions was assessed by each provincial specialists of endoscopy and statistic. The specialists of information technology were responsible for the maintenance of the database software. Finally, all data were collected in Shanghai and were totally checked again for further analysis. Consequently, if failed in any verification checks, the questionnaire would be sent back to the hospital to be revised and resubmitted until it passed.

### Statement and definition

The census was principally conducted in mainland China both in 2013 and 2020, covering 31 provincial regions (provinces, autonomous areas and municipalities). According to the current Hospitals Grading Management Rules in China,^[15]^ hospitals are classified into tertiary, secondary, primary, and ungraded from the superior to the basic. In addition, information on endoscopic ultrasonography (EUS) and colonoscopy development have been published previously in another study.^[13,16]^

In this study, volume refers to the number of cases that underwent diagnosis and treatment of digestive endoscopy. The ratio of endoscopist to volume refers to the average number of cases performed by each endoscopist, which reflects their approximate workload. The utilization rate was defined as the total volume per 100, 000 inhabitants in a certain year, which was used as the main index to assess the adequacy of each type of endoscopic procedures. In addition, the utilization rates of some developed countries were retrieved and calculated using data from published articles and a variety of electronic databases. These rates were compared to the Chinese for the same period.

Quality indicators were prospectively designed based on consensus and guidelines in China. The detection rate of early cancer was defined as the proportion of early cancer among all cancers detected during esophagogastroduodenoscopy (EGD) or colonoscopy. The malignancy detection rate (MDR) was interpreted as the detection rate of esophageal and gastric cancers during EGD (main malignant neoplasms in upper gastrointestinal tract in Chinese population). During colonoscopy, the adenoma detection rate (ADR) was determined as the ratio of the adenoma cases. For the positive rate of EUS-fine needle aspiration (EUS-FNA), if atypical or malignant cells were found in a EUS-FNA sample, the result was defined as positive. Endoscopy Quality Index (EQI), a quantitative quality evaluation tool developed by NDEIS previously, was applied to quantify the quality of endoscopy among different regions.

### Statistical analysis

Categorical data were expressed as the number of each category and its percentages. Quantitative data are expressed as median and interquartile range. A nonparametric correlation statistical test (two-sided Spearman’s test) was performed to analyze the correlations among the different parameters. Statistical analysis was performed using SPSS version 27.0 for Windows (IBM Corp., Armonk, NY, USA). Statistical significance was set at a two-sided *P* value < 0.05.

## Results

### Hospitals performing digestive endoscopy

**Figure 2:**
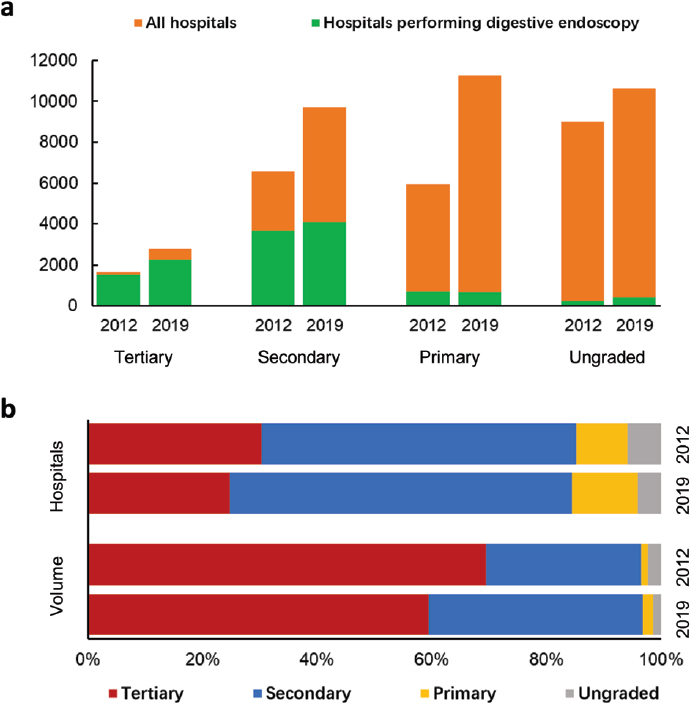
Formation of hospitals and volume for the period 2012 and 2019. (a), comparison of hospitals performing digestive endoscopy to all hospitals with different grades; (b), composition of hospitals performing digestive endoscopy with different grades; (c), composition of annual volume of hospitals with different grades.

From 2012 to 2019, the number of hospitals providing digestive endoscopy service increased from 6, 128 to 7, 470 (1.22-fold). The median hospitals per 100, 000 inhabitants per provincial region increased from 0.49 (interquartile range [IQR], 0.39–0.57) to 0.55 (IQR, 0.49–0.63). However, the percentage of these hospitals declined from 26.4% to 21.7% (Figure 2a). Nationwide, 93.5%, 55.8% and 11.8% of tertiary, secondary, and primary hospitals performed digestive endoscopy in 2012, whereas only 82.4%, 42.3% and 6.0% respectively in 2019. ^[17,18]^

Among these hospitals, the proportion of tertiary hospitals went through the most significant growth from 24.8% to 30.3% (Figure 2b). Meanwhile, more than half were secondary hospitals, despite of a decrease from 59.8% to 54.8%. Primary hospitals contributed a lower percentage ( < 10%), and ungraded hospitals remained the lowest contributors ( < 6%). The characteristics of hospitals and digestive endoscopy units were shown in Table 1.

**Table 1:** Characteristics of hospitals and digestive endoscopy units in mainland China in 2012 and 2019

	2012, *n* (%)	2019, *n* (%)
Hospital grading		
Tertiary	1,518 (24.8)	2,265 (30.3)
Secondary	3,663 (59.8)	4,097 (54.8)
Primary	704 (11.5)	676 (9.0)
Ungraded	243 (4.0)	432 (5.8)
Hospital ownership		
State-owned hospital	5,518 (90.0)	6,422 (86.0)
Private hospital	610 (10.0)	1,048 (14.0)
Hospital type		
General hospital	5,740 (93.7)	6,976 (93.4)
Specialized hospital	388 (6.3)	494 (6.6)
Affiliation of digestive endoscopy units		
Department of gastroenterology	2,908 (47.5)	3,841 (51.4)
Independent department of endoscopy	2,325 (37.9)	2,841 (38.0)
Department of general surgery	121 (2.0)	113 (1.5)
Others*	774 (12.6)	675 (9.0)
Operating room		
≤5	5,595 (91.3)	6,702 (89.7)
≥6	533 (8.7)	768 (10.3)
Area of digestive endoscopy unit (m^2^)		
≤50	2,070 (33.8)	1,391 (18.6)
51-200	2,863 (46.7)	3,441 (46.1)
201-500	789 (12.9)	1,580 (21.2)
>500	406 (6.6)	1,058 (14.2)
ERCP-operating room		
Belongs to digestive endoscopy unit	-	536 (7.2)
Share with department of radiology	-	1,054 (14.1)
Absence	-	5,880 (78.7)
System of structured graph-word report		
Yes	3,258 (53.2)	6,089 (81.5)
No	2,870 (46.8)	1,381 (18.5)
System of pathology feedback		
Yes	-	1,994 (26.7)
No	-	5,476 (73.3)

^*^Such as physical examination center, department of colorectal surgery, and outpatient department.

### Digestive endoscopists

Mainland China had 26, 203 endoscopists in 2012 and 39, 638 in 2019 (1.51-fold). Out of all physicians, endoscopists accounted for 1.06% and 1.32% respectively. The median number of available endoscopists per 100, 000 inhabitants per region was 2.8 (IQR, 2.4–3.4) in 2019, compared to 1.9 (IQR, 1.6–2.3) in 2012. The regional distribution was observed to significantly correlated with the gross domestic product (GDP) per capita in 2019 (*P* = 0.012).

Regarding the ranking, 38.2% in 2012 and 37.9% in 2019 of endoscopists were found to be attending physicians, as well as 38.7% and 39.3% were chief or associate chief physicians. Furthermore, 4, 279 (10.8%), 6, 465 (16.3%), and 4, 025 (10.2%) endoscopists could perform endoscopic retrograde cholangiopancreatography (ERCP), endoscopic submucosal dissection (ESD), and EUS respectively in 2019. More than four out of five of these advanced endoscopists were concentrated in tertiary hospitals.

### Volume and utilization rate

**Figure 3:**
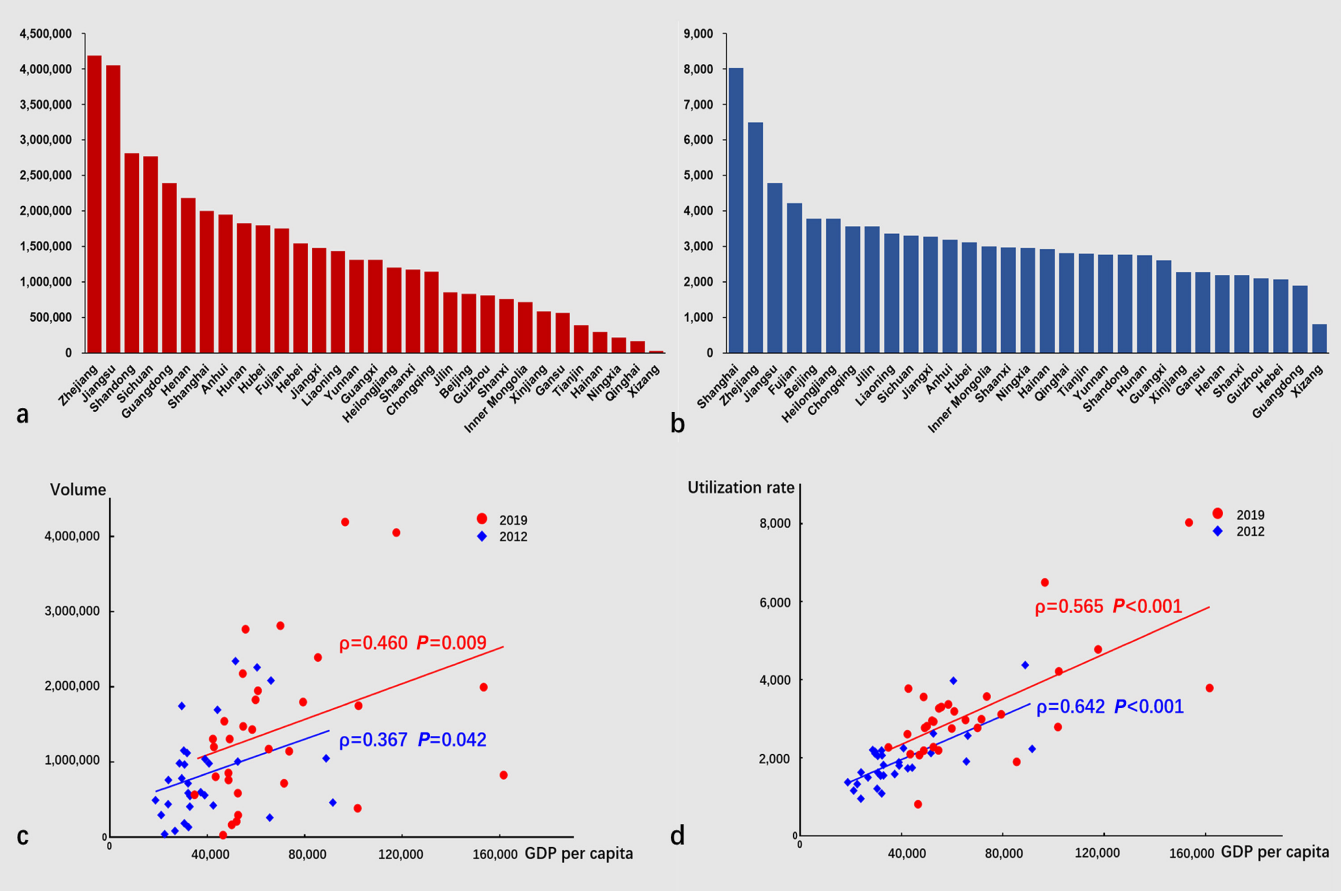
Regional distribution of annual volume, utilization rate, and their correlations with GDP per capita in mainland China. (a), annual volumes of 31 provincial regions in 2019; (b), utilization rates of 31 provincial regions in 2019; (c), scatter plot diagram showing the correlation between volume and GDP per capita in 2012 and 2019; (d), scatter plot diagram showing the correlation between utilization rate and GDP per capita in 2012 and 2019.

From 2012 to 2019, the total volume increased from 28.8 million to 44.5 million (1.55-fold). In 2019, the median volume per region was 1, 307, 524 (IQR, 719, 570–1, 948, 522) and totally 19.6 million cases (44.1%) were performed under sedation or anesthesia. Nationwide, there was a substantial disparity in regional distribution (Figure 3a), and a significant correlation were observed between the volume and GDP per capita (*P* < 0.05) (Figure 3c). Details of the different types of endoscopic procedures are demonstrated in Table 2.

**Table 2:** The volume (*10,000) of different digestive endoscopic procedures in 2012 and 2019

Procedures	2012	2019 (growth rate, %)
Esophagogastroduodenoscopy	2225.41	2937.33 (32.0)
-under anesthesia/sedation	-	1229.76
Colonoscopy	583.24	1291.55 (121.4)
-under anesthesia/sedation	-	683.37
Endoscopic resection		
Polypectomy	-	396.66
EMR	-	118.37
ESD	9.67	18.97 (96.2)
-of esophagus	2.10	3.61 (71.9)
-of gastric	4.16	9.71 (133.4)
-of colorectum	3.14	11.44 (264.3)
STER	-	0.96
ERCP	19.56	20.05 (2.5)
EUS^*^	20.72	46.42 (124.0)
-of gastrointestinal tract	-	38.15
-of biliary tract and pancreas	-	6.73
-interventional EUS	1.07	1.53 (43.0)
Balloon-assisted enteroscopy	2.64	3.24 (22.7)
Capsule endoscopy	-	7.68
Capsule endoscopy of small-bowel	4.84	5.46 (12.8)
Magnetically controlled capsule gastroscopy	-	2.22
Other endoscopic procedures^#^	-	63.76

EMR, endoscopic mucosal resection; ESD, endoscopic submucosal dissection; STER, submucosal tunnel endoscopic resection; ERCP, endoscopic retrograde cholangiopancreatography; ^#^Other endoscopic procedures include: endoscopic hemostasis for non-variceal upper gastrointestinal bleeding, endoscopic removal of foreign body, stricture dilation/stent placement, photodynamic therapy, endoscopic variceal ligation/endoscopic variceal sclerosis, peroral endoscopic myotomy, radiofrequency ablation (RFA), percutaneous endoscopic gastrostomy/percutaneous endoscopic jejunostomy, and needle RFA of gastroesophageal reflux disease. ^*^EUS data have been previously reported.

Overall, 59.5% and 69.4% of the volume was generated by tertiary hospitals, while 37.3% and 27.1% was generated in secondary hospitals respectively in 2012 and 2019. Only a tiny minority was added by primary and ungraded hospitals (totaling less than 4%) (Figure 2c). Furthermore, the ratio of endoscopists to volume increased from 1: 1098 in 2012 to 1: 1123 in 2019.

**Figure 4:**
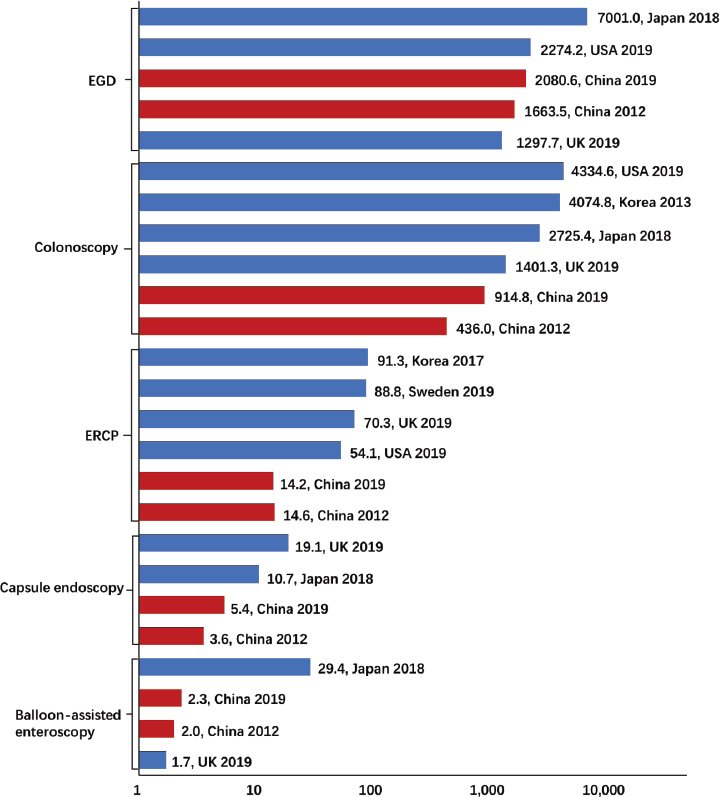
Utilization rates of different types of procedures in mainland China and other developed countries. (USA, the United States of America; UK, the United Kingdom. Some of Japanese volumes were from NDB open data. The response rate of UK endoscopy census was 68.4%. The EUS rates of China and developed countries had been published previously.)

The median utilization rate per region was 1878 (IQR, 1520.7–2209.8) in 2012 and 2953.8 (IQR, 2275.8–3558.3) in 2019. The regional utilization rate also showed a significant correlation with the GDP per capita (Figure 3b, 3d). During the corresponding period, the utilization rates of several main digestive endoscopic procedures in China were much lower than those in some developed countries (Figure 4).^[3,4,19,20,21,22]^

### Quality of digestive endoscopy

**Figure 5:**
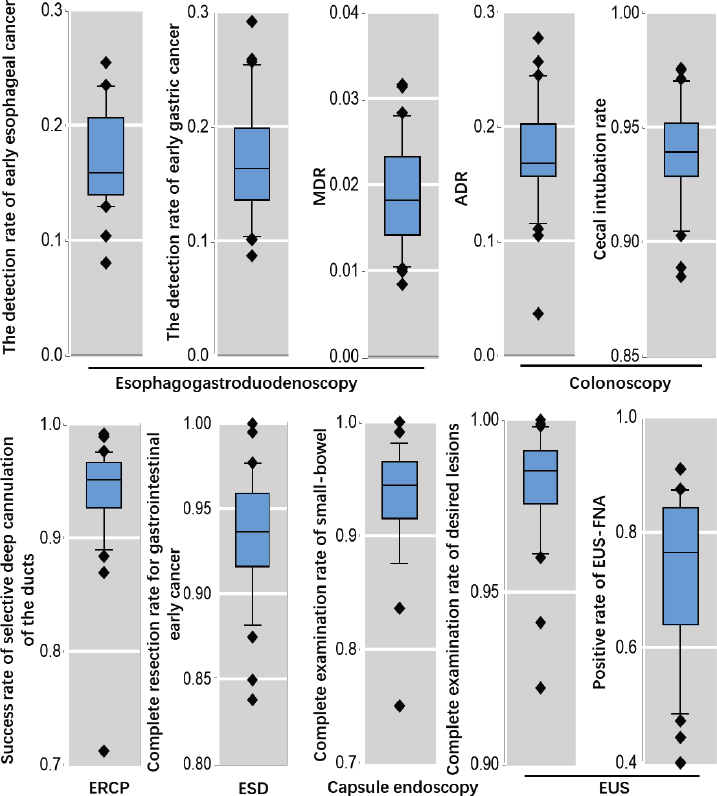
Distribution of ten results of quality indicators in 31 regions across mainland China in 2019.

In 2020, data regarding ten quality indicators were collected, concerning six predominant procedures of digestive endoscopy. The detection rates of early esophageal and gastric cancers were 18.6% and 17.2% respectively, and the MDR during EGD was 1.9%. The ADR and cecal intubation rate for colonoscopy were 18.1% and 94.4%, respectively. The success rate of selective deep cannulation of the ducts for ERCP was 93.6%. The complete resection rate for early gastrointestinal cancer during ESD was 93.57%. The complete examination rate of the small-bowel during capsule endoscopy was 93.66%. As for EUS, the rate of complete examination of desired lesions and the positive rate of EUS-FNA was 98.05% and 77.55%, respectively. Nationwide, these qualities varied considerably from region to region (Figure 5). The average standardized EQI was 647.7 (SD, 76.5), and significant variations were observed. By analysis, we identified that the EQIs were positively correlated with regional GDP per capita (*P* < 0.01), but they showed no correlations with utilization rates, hospitals and available endoscopists per 100, 000 inhabitants (*P* > 0.05).

## Discussion

To the best of our knowledge, this is the most recent and detailed study conducted before the outbreak of COVID-19. Since 2020, it was reported that digestive endoscopy in many countries has suffered dramatic decreases in procedural volume or cancer screening and increases in the burden of endoscopy training.^[23,24,25,26,27,28,29]^ The international consensus and statements produced a series of best practice recommendations and strategies in pursuit of the orderly recovery.^[30, 31]^ Exactly, our study represents a milestone in development under normal trends. In the aftermath of China’s staged victory regarding pandemic prevention and control, this evidence was essential for further improvement and regulation towards provision of better services for the entire population.

In this study, the status quo and development of digestive endoscopy was analyzed in the largest developing country with one-fifth of the world population. The problems uncovered could be of great use to policymakers so that they can identify areas that need strengthening. There are also some help and insights for other developing countries regarding health policy and the arrangement of resources. At the same time, it provides a reliable reference for domestic and international scholars for further study.

Hospitals, endoscopists, volumes, and qualities were carried to new and higher levels across the country. Meanwhile, advanced endoscopies and many emerging endoscopic technologies have achieved initial success, such as ESD and radiofrequency ablation. On the one hand, economic and technological development are largely responsible for this progress. An increasing number of people are paying more attention to their digestive health and can afford better healthcare services. On the other hand, changes in diet, stress, and lifestyle have led to a heavier burden of digestive system disease in China. Over the past, mortality due to gastrointestinal cancer has remained stubbornly high and the mortality to incidence rate has even ascended.^[32,33]^ More importantly, this stride originated from national policies and efforts for Universal Health Coverage in China over the past decades, such as the Healthy China strategy, healthcare reform, and popularization of health and health insurance of inhabitants.^[34,35]^

However, our findings raise some issues for timely reflection. Firstly, the priority of development was neglected. Digestive endoscopy has been lagging in the entire development of medical and health services. In addition, it is important to note that the resources were over-concentrated in tertiary hospitals. The overwhelming majority of primary hospitals did not provide digestive endoscopy service. This was found to be true for other primary healthcare institutions, including community health centers in urban areas, township health center in rural areas, and kinds of clinics. But in China, these are superior in quantity and convenience. To some extent, this was consistent with the challenges facing healthcare system,^[36]^ and it implied that digestive endoscopy was not adequately popularized in China. To provide more effective service, the emphasis should be shifted towards primary healthcare. Otherwise, it could not serve the health needs of the accelerating aging population with an increasing burden of digestive disease. Currently, NDEIS and NHC have been committed to developing secondary hospitals, mainly including county- and district-level hospitals, which is considered a transition stage to provide all inhabitants with affordable and basic services.

Secondly, the utilization rates of certain procedures were lower than those of developed countries, which indicated considerable insufficient service against desperate needs. We not only need to explore advanced endoscopic techniques, ^[37, 38]^ but also pay attention to use of the basic ones. The most conspicuous disparities were observed for EGD and colonoscopy, although they experienced a higher growth rate in China. Gastrointestinal cancer remains a significant regional health problem all over the world. Currently, the age-standardized incidence rates of colorectal, gastric, and esophageal cancers were 23.9, 20.6, and 13.8 per 100, 000 in China, 38.5, 31.6, and 7.2 in Japan, and 25.6, 4.2, and 2.8 in the United States, respectively.^[39]^ However, Japan and the United States performed better in adequacy, which was largely owing to their reasonable endoscopic cancer screening. As a developing country, China is relatively scarce in medical resources. To overcome this shortcoming, more attention should be paid to the effective use of limited resources. A national program for early diagnosis and treatment of gastrointestinal cancer was launched for the target population, which was aimed at ameliorating the prognosis of patients and alleviating the familial or governmental strain of medical costs. Next, we aimed to establish a more comprehensive system based on the regional incidence, endoscopy quality, exposure to risk factors, and economics to guide endoscopic screening more precisely.

Thirdly, further efforts are still necessary to improve quality. For example, the detection rates of early esophageal and gastric cancers were 75.8% and 51.6% in Japan,^[40]^ and the ADR were 28.7% in Japan and 38.1% in the United States respectively.^[41,42]^ Those results are higher than the Chinese. However, it is noteworthy that the Chinese results came from national census, which cannot literally interpret the quality because of different baseline populations. In contrast to excellent endoscopic screening, many Chinese patients do not undergo examinations until they show symptoms of advanced disease. As a result, quality indicators are reduced and medical poverty is created. Instead, certain results in China have approached or even exceeded the level of developed countries. For instance, overall cecal intubation rate of colonoscopy and complete examination rate of capsule endoscopy were 94.4% and 93.66% respectively, while 93.1% and 83.7% in Korea.^[43,44]^ The success rate of selective deep cannulation during ERCP was 95.67%, and this rate was 93.1% in Sweden in the same year.^[20]^ Besides, based on the previous results from 5-year consecutive nationwide surveys and multicenter prospective studies,^[45]^ the success rate of extraction of common bile duct stones ( < 1 cm) and proportion of pancreatitis after ERCP were 94.2% and < 10% respectively. These achievements have indeed encouraged the Chinese digestive endoscopy. In the future, we think very highly of artificial intelligence (AI), which will create an additional shortcut for Chinese digestive endoscopy.

Fourthly, the work circumstances of Chinese endoscopists were bleak. The average number of available endoscopists was now far below demand and their workload was even higher. An excessive number of patients on hold arose from severe time and energy constraints, especially endoscopists in tertiary hospitals. This also halted the improvement of quality. Currently, there are widespread trainings of professional endoscopists in China to improve their competence and academic qualifications. In addition, an AI-assisted prediction tool with favourable performance for screening of oesophageal squamous cell carcinoma and adenocarcinoma of the oesophagogastric junction was developed to prevent the need for endoscopy screening in many low-risk individuals and ensure resource optimisation by prioritising high-risk individuals in community. ^[46, 47]^ This could greatly reduce the burden of endoscopists in cancer screening.

Finally, regional inequality is a serious problem. Income-related inequality was not only seen in hospitals and endoscopists, but also in volume and utilization rates. Over the past seven years, these health inequalities have not narrowed. Taking utilization rates as an example, they are thought to be closely related to the incidence of digestive cancers. But the rates of many provinces with high incidence were lower than those of others. The contradiction between the unbalanced development and the increasing demand for cancer screenings makes policy- and hospital-level actions imminent in China. Moreover, the overall quality of endoscopy was unbalanced. Regions with higher economic level got better results in EQIs, which largely meant that economic development came first.

Our study has several limitations. First, recall bias cannot be avoided because of its retrospective design. Second, the census data were submitted by online questionnaire, which may have affected the accuracy. Because of these issues, the authenticity and reliability of some data need to be improved. In the future, a national prospective registry system to directly capture quality control data from various medical institutions is necessary. Finally, the utilization rates of other developed countries were estimated from studies and reports with different designs and coverage. There was inevitable variance in the indication, specific technique, and population structure among these countries. Therefore, the comparison just drew an outline.

In summary, extraordinary progress and visible challenges coexisted in digestive endoscopy in China, which rooted in stage of developing world and were inevitable to be addressed in the future. With the success of the 20th National Congress of the Communist Party of China, the idea and will of improving people’s health have reached a new and higher level. Our belief is that the Chinese digestive endoscopy will yield more social benefits in the way to Healthy China 2030.
